# Similar herpes zoster incidence across Europe: results from a systematic literature review

**DOI:** 10.1186/1471-2334-13-170

**Published:** 2013-04-10

**Authors:** Sybil Pinchinat, Ana M Cebrián-Cuenca, Hélène Bricout, Robert W Johnson

**Affiliations:** 1 Biostatem, Parc d’Activité Via Domitia, 205 Avenue des Gardians, Castries, 34160, France; 2 Centro de Salud de Ayora, Avenida Argentina, Ayora, Valencia, 7 46620, Spain; 3 Epidemiology Department, Sanofi Pasteur MSD, 8 rue Jonas Salk, Lyon, 69007, France; 4 Department of Ophthalmology, University of Bristol, Bristol, UK

**Keywords:** Epidemiology, Herpes zoster, Shingles, Europe, Incidence

## Abstract

**Background:**

Herpes zoster (HZ) is caused by reactivation of the varicella-zoster virus (VZV) and mainly affects individuals aged ≥50 years. The forthcoming European launch of a vaccine against HZ (Zostavax®) prompts the need for a better understanding of the epidemiology of HZ in Europe. Therefore the aim of this systematic review was to summarize the available data on HZ incidence in Europe and to describe age-specific incidence.

**Methods:**

The Medline database of the National Library of Medicine was used to conduct a comprehensive literature search of population-based studies of HZ incidence published between 1960 and 2010 carried out in the 27 member countries of the European Union, Iceland, Norway and Switzerland. The identified articles were reviewed and scored according to a reading grid including various quality criteria, and HZ incidence data were extracted and presented by country.

**Results:**

The search identified 21 studies, and revealed a similar annual HZ incidence throughout Europe, varying by country from 2.0 to 4.6/1 000 person-years with no clearly observed geographic trend. Despite the fact that age groups differed from one study to another, age-specific HZ incidence rates seemed to hold steady during the review period, at around 1/1 000 children <10 years, around 2/1 000 adults aged <40 years, and around 1–4/1 000 adults aged 40–50 years. They then increased rapidly after age 50 years to around 7–8/1 000, up to 10/1 000 after 80 years of age. Our review confirms that in Europe HZ incidence increases with age, and quite drastically after 50 years of age. In all of the 21 studies included in the present review, incidence rates were higher among women than men, and this difference increased with age. This review also highlights the need to identify standardized surveillance methods to improve the comparability of data within European Union Member States and to monitor the impact of VZV immunization on the epidemiology of HZ.

**Conclusions:**

Available data in Europe have shortcomings which make an accurate assessment of HZ incidence and change over time impossible. However, data are indicative that HZ incidence is comparable, and increases with age in the same proportion across Europe.

## Background

Varicella-zoster virus (VZV) is a herpes virus that infects nearly all humans and causes two distinct diseases: varicella, the primary infection which usually occurs in childhood, and herpes zoster (HZ), a result of reactivation of VZV which remains latent in the sensory ganglia following varicella. This reactivation occurs when VZV-specific cellular-mediated immunity decreases, mainly due to age-related immunosenescence and immunosuppressive conditions.

HZ is characterized by a vesicular skin rash localized in the sensory region of the affected ganglia, and is often preceded, or accompanied by acute pain or itching. The individual lifetime risk of developing HZ is between 23.8% and 30%, or approximately 1 in 4 people [1-5]. However, for individuals aged 85 and over, this risk increases to 1 in 2 people [6]. Indeed, HZ incidence increases markedly after 50 years of age, with two-thirds of HZ cases occurring in individuals aged 50 years or over [7]. Anyone who has had varicella is at risk of HZ, and in Europe varicella affects over 90% of children before the age of 15 years [8].

HZ is painful during the acute phase, but pain may persist for months or even years. Post-herpetic neuralgia, defined as chronic pain persisting after rash onset, occurs in 20% to 50% of patients, and can lead to several months of treatment and loss of quality of life [9,10]. After 1 year, almost 10% of patients, mainly older people, still have persistent pain [11,12].

The forthcoming European launch of a vaccine against HZ (Zostavax®) prompts the need for a better understanding of the epidemiology of HZ in Europe. Therefore the aim of this review was to summarize the available data on HZ incidence in Europe and to describe age-specific incidence, notably among individuals aged over 50 years.

## Methods

### Literature search

The Medline database of the National Library of Medicine was used to conduct a comprehensive literature search of population-based studies of HZ incidence published between 1960 and 2010. Articles had to include the MeSH term “herpes zoster” or “shingles”, as well as the keyword “incidence” or “age-specific incidence”. Only articles reporting on studies carried out in the 27 European Union Member States, Iceland, Norway, or Switzerland (complete list can be found at end of this paper) were considered. Publications in Dutch, English, French, German, Italian or Spanish were considered.

References lists from retrieved publications were also checked manually for any additional studies or review articles on the epidemiology of HZ, and if necessary the authors were contacted to obtain data on age-specific HZ incidence rates. National surveillance data, the websites of the National Institutes of Health of the United Kingdom (UK), sentinel networks and data from the World Health Organization were also consulted, especially for countries where no publications were found. However, no additional data were identified.

### Selection criteria

Included articles had to have HZ incidence data available, a population-based study design and information on the quality criteria used to score the studies in this review. Any study that did not contain this information was excluded.

All studies limited to immunocompromised populations/populations with primary or acquired immunodeficiency status, patients with hematological malignancies (acute and chronic leukemia, lymphoma or other malignant neoplasm affecting the bone marrow or lymphatic system, solid tumors receiving cytotoxic chemotherapy, hematopoietic stem cell transplantation), persons with AIDS, patients on immunosuppressive therapy (i.e., treatment with agents, such as x-rays, corticosteroids, or cytotoxic chemicals, etc.) were excluded. A few articles were also excluded due to duplicate publication, or lack of study dates.

### Quality assessment and scoring of articles

In order to provide a methodological classification of the studies, a reading grid was created specifically for this review based on set quality criteria. According to these criteria, each selected article was scored by two independent readers. The reading grid allowed for a total of 30 possible points:

1) Representativeness of the sample of patients (12 points): number of practitioners or specialists, sampling method description and validation—if any, geographic distribution, population covered.

2) Incidence calculation (12 points): estimation of the denominator used to calculate incidence, presence of confidence intervals, estimation of age- or sex-specific incidence, size of the study, diagnostic criteria of HZ.

3) Study design (4 points):

a) Prospective inclusion of patients (considered high-quality data) suffering from HZ in health care facilities during a defined study period, either during an ad-hoc study or through a sentinel surveillance network.

b)) Retrospective identification of HZ cases either through the review of medical files in a sample of practitioners, or through the analysis of large databases (national registries, health insurance databases, etc.). In the first case, potential issues could arise from the quality of files and missing data. Database studies are less time- and cost-consuming for assessing incidence rates; however these studies are subject to bias related to the completeness of the database and inference to the general population.

4) Discussion of the study limitations and study results put in perspective with the data from the literature (2 points).

Papers with a quality score of less than 15 out of the 30 possible points were excluded from the literature review. For the selected articles, pertinent information was extracted, including study dates, setting, study population, sample size, diagnostic criteria used for HZ, overall HZ incidence with 95% confidence intervals (when available), and incidence data by age, and by sex (when available).

The present systematic review is following the PRISMA guidelines [13].

## Results

The Medline search identified 1 644 articles, of which 1 563 were immediately excluded based on their abstracts. A review of the reference lists of the 81 remaining articles identified 23 additional articles, making a total of 104. Of these, 77 were excluded due to the inclusion and exclusion criteria: four hospital-based studies, six cost-effectiveness studies (some epidemiological data, but obtained or derived from several community-based studies), 34 general reviews, 18 studies in countries not included in this review, and 15 with no exploitable incidence data or that lacked information on the quality criteria necessary to score the publication.

**Figure 1:**
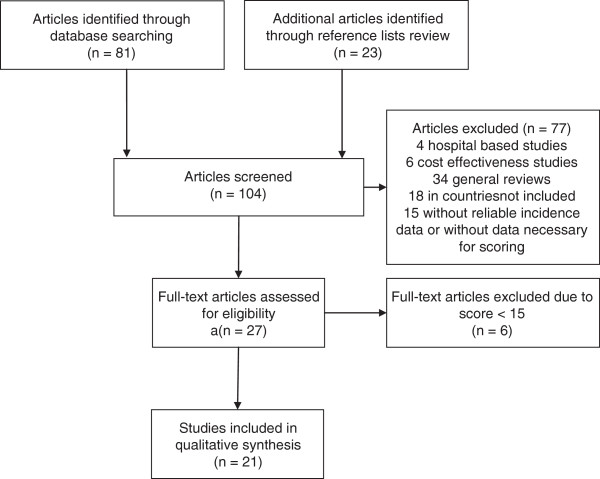
Flow diagram.

The 27 remaining articles that corresponded to the inclusion criteria were scored using the reading grid. After the reading grid was applied, six of the 27 studies were further excluded as they did not meet the threshold for inclusion (i.e., 15 points) [14-19]. Therefore, 21 articles were finally included in this review (Figure 1).

The 21 reports of HZ incidence from European countries included one from Belgium, four from France, two from Germany, two from Iceland, two from Italy, three from the Netherlands, two from Spain, one from Switzerland, and four from the UK (Table 1). No relevant data were found for the other 21 countries considered in this review (Austria, Bulgaria, Cyprus, the Czech Republic, Denmark, Estonia, Finland, Greece, Hungary, Ireland, Latvia, Lithuania, Luxembourg, Malta, Poland, Portugal, Romania, Slovenia, Slovakia, Sweden and Norway).

**Table 1:** Selected details of included studies

	**Ref**	**Country**	**Study dates**	**Author**	**Design**^**a**^	**Concerned population**	**Age criteria**	**Case ascertainment**	**Diagnosis**	**Cases (n)**	**Incidence /1 000 PY [95% CI]**^**b**^
1	[20]	Belgium	1994-03	Truyers	A_2_	Patients of 51 GPs	All	Notified by GPs	ICPC-code S70	NR	4.57 [4.31-4.79]
2	[21]	France	2009	Sentinel network (INVS)	A_2_	454 active GPs (62 809 976 patients)	All	Weekly returns of all diagnoses	Individual GPs	992	5.52 [5.06-5.98]
3	[22]	France	2005-08	Gonzales-Chiappe	A_2_	Patients of ~1200 GPs	All	Notified by GPs	Individual GPs	2375	3.82 [3.64-4.05]
4	[23]	France	2005	Mick	B_1_	Patients of 231 GPs, 41 dermatologists, 15 neurologists	≥50y	Postal survey – cases seen in 2005	Individuals clinicians	777	8.99 [8.34-9.64]
5	[24]	France	1998	Czernichow	B_1_	Patients of 744 GPs	All	Postal survey of GPs – cases seen in previous year	Individual GPs	605	3.20 [3.00-3.40]
6	[25]	Germany	2004	Schiffner-Rohe	B_2_	120 399 patients	≥50y	Searches in computerized records	ICD-10	1176	9.80 [9.20-10.40]
7	[26]	Germany	1992-93	Paul	A_1_	Population of Ansbach City (about 40 000 inhabitants)	All	All cases seen by GPs, dermatologists, pediatricians	Individual clinicians	152	2.26
8	[27]	Iceland	1990-95	Helgason	A_1_	Patients of 62 GPs (out of 150 GPs in Iceland)	All	Notified by GPs	Individual GPs	462	2.00 [1.80-2.20]
9	[28]	Iceland	1990-95	Petursson^c^	A_1_	Patients of 62 GPs	<20y	Notified by GPs	Individual GPs	118	1.60
10	[29]	Italy	2003-05	EmbertiGialloreti	B_2_	Patients of 342 GPs (0.8% of Italian GPs) (450 000 patients)	≥15y	Searches in computerized records	ICD-9	5675	4.31 [4.11-4.52]
11	[30]	Italy	2004	Di Legami	A_1_	Population of Piemonte (26 934 patients)	≥14y	Notified by GPs	Individual GPs	46	1.74 [1.28-2.32]
12	[31]	Netherlands	2001	Opstelten	A_1_	104 GPs (about 390 000 patients)	All	Notified by GPs	Individual GPs	1080	3.20 [3.00-3.40]
13	[32]	Netherlands	1998-01	de Melker	B_1_	Patients of 43 GPs (about 1% of the Dutch population)	All	Notified by GPs	Individual GPs	NR	3.25
14	[33]	Netherlands	1994-99	Opstelten	B_2_	22 GPs in six areas (about 49 000 patients)	All	Searches in computerized records	Individual GPs	837	3.40 [2.90-3.90]
15	[34]	Spain	2007	Cebrian-Cuenca	A_1_	24 GPs in Valencia community (about 36 030 patients > 14y)	>14y	Notified by GPs	Individual GPs	146	4.10 [3.40-4.70]
16	[35]	Spain	2005-06	Garcia-Cenoz	B_2_	Patients of GPs in Navarre	All	Searches in computerized records	ICD-10	4959	4.15
17	[36]	Switzerland	1998-01	Richard	A_2_	Patients of 250 physicians (GPs, pediatricians, physicians of internal medicine)	All	Notified by physicians	Individual clinicians	2236	2.36
18	[37]	UK	2000-06	Gauthier	B_2_	603 GPs Research Database (GPRD) (3 million of patients)	≥50y	Searches in computerized records	ICD-10, first episode	25 002	5.23 [2.17-5.29]
19	[38]	UK	1947-72	Hope-Simpson	B_1_	GPs in Cirencester (about 3700 patients)	All	All recorded cases	NR	321	3.40
20	[39]	UK	1994-01	Fleming	B_2_	Up to 91 GPs (RCGP) (About 200 000 patients)	All	Weekly returns of all diagnoses	Individual GPs	14 532	3.20
21	[40]	UK	1991-00	Brisson	B_2_	69 GPs in England & Wales (~570000 patients)	All	Searches in computerized records	ICD-9	112 409	3.73

*Abbreviations: PY* person-years, *CI* confidence interval, *NR* not reported, *GP* general practitioner, *ICD-10* International Classification of Diseases, version 10, *UK* United Kingdom.

^a^ Design:

A. Prospective study: A_1:_ ad hoc health care-based study A_2_: sentinel practice network-based study.

B. Retrospective study: B_1_:ad hoc study with review of patients’ medical charts B_2_: database study.

^b^ For some studies, the confidence interval of incidence is not specified.

^c^ The Petursson study is a sub-analysis of the Helgason study among children and adolescents aged less than 20 years.

**Figure 2:**
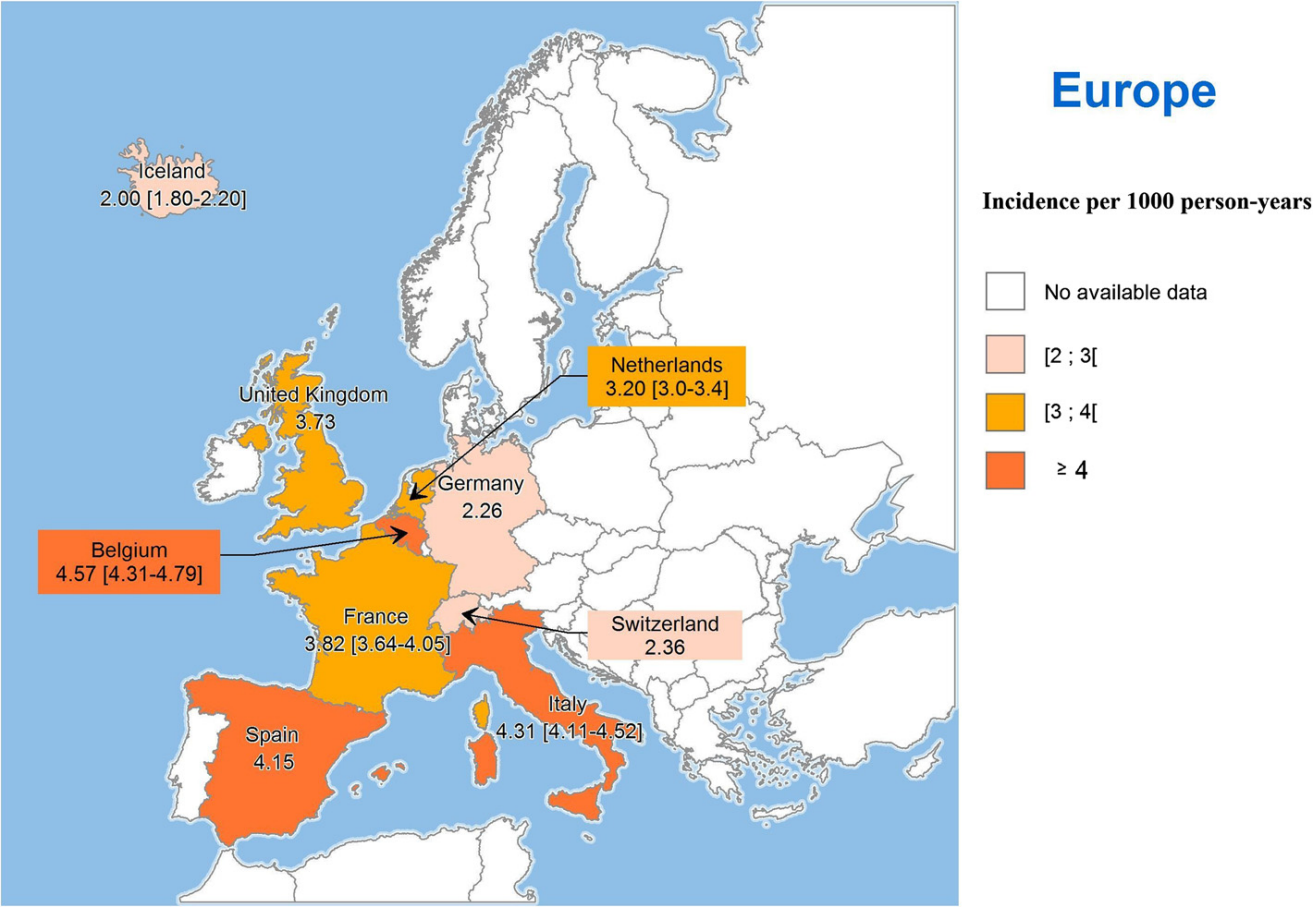
**Overall annual herpes zoster (HZ) incidence rates in Europe (/1 000 person-years).** Notes: The confidence interval is presented when available in the original publication. In case of several publications per country, the publication with the most recent data and that reported the overall HZ incidence rate is depicted.

Table 1 shows the main features of the included studies and HZ incidence by country. Annual HZ incidence varied by country from 2.0 to 4.57 per 1 000 person-years (PY). The HZ incidence rates in the studies with a score inferior to 15 were in the same range (from 3.2 and 4.14 per 1000 person-years) [15,16]. The overall incidence was lower in Iceland, Germany and Switzerland (around 2/1 000 PY), medium in the UK, the Netherlands and France (around 3/1 000 PY), and higher in Belgium, Spain and Italy (around 4/1 000 PY) (Table 1). However, no geographic trend of overall incidence was clearly observed (Figure 2).

It was estimated that in England and Wales alone there are approximately 225 000 new cases of HZ each year [40]. In 2009, the French sentinel network estimated that there were around 350 000 cases of HZ across all age groups [21]. Another study performed in France reported around 182 500 incident cases among immunocompetent people aged 50 years or over [23].

Using the nine most recent studies, which had the highest quality score for their country and were performed without age criteria [20,22,26,29,31,35,36,40],[41], we estimated an average HZ incidence rate of 3.4 ± 0.2/1 000 for all age groups combined. If this is applied to the total European population of 512 million inhabitants [42], a rough estimate of 1.7 ± 0.1 million new HZ cases can be expected each year in Europe.

**Figure 3:**
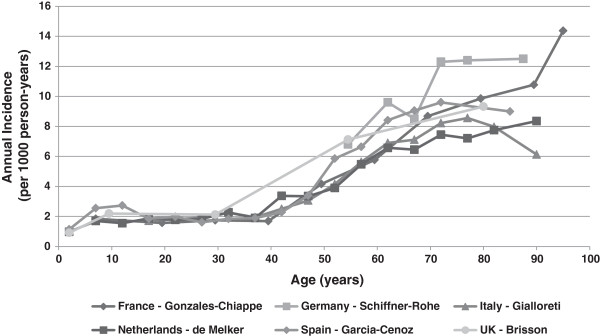
**Herpes zoster incidence by age in Europe.** Note: These studies were the most recent with available HZ incidence data by age group per country.

Eleven publications from seven countries presented both overall and specific incidence rates by sex and/or by age group (See Additional file 1). In spite of the age groups, which differed from one study to another, age-specific HZ incidence rates appeared to hold steady during the review period at around 1/1 000 children <10 years, around 2/1 000 adults aged < 40 years, around 1–4/1 000 adults aged 40–50 years, and then increased rapidly after 50 years to around 7–8/1 000, up to 10/1 000 at 80 years of age and older (Figure 3). Figure 3 illustrates that in many countries in Europe, HZ incidence increases with age, and quite steeply so after 50 years of age. In all studies included in this review, incidence rates were consistently higher among women than men (male/female ratio range: 1.13–1.56), and this difference also increased with age.

Studies performed among immunocompetent people and among the general population (including both immunocompetent and immunocompromised people), showed that the risk of HZ was higher in the general population (9.80/1 000 in Germany and 4.31/1 000 in Italy) than among immunocompetent people (9.50/1 000 (Germany) and 4.07/1 000 (Italy)) [25,29].

## Discussion

The present literature review of HZ incidence in Europe showed similar HZ incidence across the included countries for which data was available. Overall annual HZ incidence varied from 2.0–4.6/1 000 PY depending on the country, which is consistent with previous published estimates [43], and similar to those published in North America (1.25–3.7/1 000 PY [44,45]).

Our review confirms that HZ incidence increases sharply with age, from around 1/1 000 children <10 years up to 10/1 000 people over 80 years of age. These results are consistent with recent published estimates by Volpi *et al*. [43]. Annual HZ incidence in Europe has been reported as 0.3–0.74/1 000 children <10 years, 1.6/1 000 adults aged <40 years, 2.5/1 000 adults aged 20–50 years, 7.8/1 000 adults aged 60 years or over, and 10/1 000 in elderly adults over 80 years of age [43].

As expected, the same increase in incidence rates with age was observed in the studies included in this review that reported age-specific incidence (Figure 3). The correlation between age and HZ incidence may be related to a decreased cellular-mediated immune response to VZV as result of immunosenescence [44,46]. It has been suggested that exposure to varicella reduces the risk of VZV reactivation by boosting specific immunity to the virus [38,47]. This hypothesis is supported by some studies which showed that repeated familial or occupational exposure is associated with a reduced risk of HZ [48-51], but others did not confirm this [46,52].

This review showed that incidence rates are systematically higher among women than men (male/female ratio around 1.4), and this difference increases with age, which has also been found in other studies [41,53]. Women over 50 years of age seem to be particularly at risk. However, it is unclear whether the risk of HZ is increased in all women. Women might simply be more likely to seek medical advice, thereby causing a higher reporting rate, or there may be some biological mechanism by which women are more susceptible to VZV reactivation [54].

Our review excluded studies limited to immunocompromised populations, or individuals with primary or acquired immunodeficiency status. Nevertheless, as included studies were population-based, some of them made a distinction between the total study population and the immunocompetent population [25,29]. This review confirms that immunocompetent patients are at lower risk of developing HZ than the general population [25,29]. The control of VZV reactivation depends on the maintenance of adequate levels of cellular-mediated immunity to VZV, which explains why cellular-mediated immune deficiency is a risk factor for developing HZ [54].

In Europe, not all countries have some form of surveillance in place for HZ [55,56] and there is marked heterogeneity in the type of HZ surveillance systems that do exist (national mandatory or sentinel), the type of data collected (case-based or aggregated) and the reported case classification (clinical and/or laboratory) [57]. Most surveillance systems operate using reports of clinical cases [57].

This review highlights the need to identify standardized surveillance methods in order to improve data comparability within European Union Member States and, in the framework of introducing HZ vaccination, to monitor the impact of immunization on the epidemiology of HZ.

Since most of the European studies in this review were performed and published in the last 10 years, it was difficult to look at a time trend variation in the risk of HZ. The only country (the UK) with two incidence rate estimates, which were about 30 years apart, provided two close figures: 3.40/1 000 people in 1975 *vs.* 3.73/1 000 people in 2000 [38,40]. However, this comparison is delicate since the first study was retrospective [38] and the second prospective [40].

In the literature, there are conflicting data with regard to whether age-adjusted HZ incidence is changing over time [7,58]. Indeed, the literature fails to show evidence of any change of HZ incidence over time, notably in relation to varicella vaccination. Longitudinal data, including a few years of baseline before possible routine use of the varicella vaccine in children or adolescents, and a sufficient number of years of data to detect a trend (at least 3, preferably more) after the implementation of the vaccine will be needed to assess the impact of varicella vaccination on HZ incidence [59]. Such data are available from the US where varicella vaccination has been used routinely since 1995; however no clear conclusions were drawn on the impact on HZ incidence. Some authors did not observe any impact of varicella vaccination on HZ incidence [7,58] and others observed an increase [60]. Moreover, looking at a potential HZ incidence trend overtime is challenging and depends on the availability of baseline data collected using comparable study methods in populations with comparable health care behavior. Comparing results across studies and time periods must take into account different study methods and must adjust for changes in the age structure of the population over time. As the proportion of older people grows in Europe [61], HZ is likely to become a more important public health issue in the future. The apparently increasing proportion of immunocompromised persons due to medical conditions or medication in the population, and the effect this may have on HZ, must also be considered.

This literature review has various limitations. First of all, this review included studies with different designs: direct prospective recruitment of patients with HZ in health care settings during a defined study period, and retrospective identification through medical files from a number of practitioners. In general, prospective recruitment methods are considered to be preferable, whereas retrospective recruitment poses some methodological problems regarding data quality and missing data. However, this was taken into account in the reading grid, which assigned a higher quality score to prospective studies than retrospective studies.

Moreover, in spite of their potential shortcomings, some studies based on large databases (UK, Italy, Spain, Germany) were included in this review. It is true that in the past the methods used in population-based studies, such as those used to extrapolate results obtained from a single database to the entire North American population, have been criticized [62]. Indeed, in this case the fact that the denominator used was the total number of persons registered in the national health care system and was presented as exhaustive raised a methodological problem linked to the calculation of the HZ incidence rate. This was questionable since no information was given on the number of persons who were not registered, compared to the national census. In that case, the denominator was a surrogate for the true number and the calculated rate could have been over-estimated. Recently, Yawn *et al.* showed that administrative data use alone appears to overestimate the number of HZ cases [53], and the potential coding error of HZ diagnosis in administrative data has also been investigated [63].

## Conclusions

Available European epidemiological data on HZ have shortcomings which make accurate assessment of HZ incidence and change over time impossible. However, data are indicative that HZ incidence across Europe is comparable (about 3.4 ± 0.2/1 000 when considering all age groups) and increases with age, especially after 50 years of age. This equates to a total of 1.7 +/− 0.1 million new HZ cases each year in Europe.

### Complete list of countries

Austria, Belgium, Bulgaria, Cyprus, Czech Republic, Denmark, Estonia, Finland, France, Germany, Greece, Hungary, Iceland, Ireland, Italy, Latvia, Lithuania, Luxembourg, Malta, the Netherlands, Norway, Poland, Portugal, Romania, Slovakia, Slovenia, Spain, Sweden, Switzerland, United Kingdom.

## Abbreviations

HZ: Herpes zoster; PY: Person-years; UK: United Kingdom; VZV: Varicella-zoster virus

## Competing interests

This study was funded by SPMSD.

SP is an employee of Biostatem, Castries, France. She had specified relationships with Sanofi Pasteur MSD that might have an interest in the submitted work in the previous 6 years.

HB is an employee of SPMSD, Lyon, France.

AC declares that she has no competing interests.

RJ has received honoraria for consultancy, lectures and scientific meeting attendance from Sanofi Pasteur MSD, Merck Inc., Merck Frosst, Novartis and Astellas.

## Authors’ contributions

SP carried out the literature search, the selection of the articles, the scoring of the selected papers, and the extraction of the data and drafted the manuscript. HB performed the scoring of the selected papers, the extraction of the data and participated in drafting the manuscript. AC and RJ helped to interpret the results of the review and to draft the manuscript. All authors read and approved the final manuscript.

## Pre-publication history

The pre-publication history for this paper can be accessed here:

http://www.biomedcentral.com/1471-2334/13/170/prepub

## Supplementary Material

Additional file 1 **Incidence rates of herpes zoster (HZ) by age group and by sex when available (/1 000) **[20,22,23,25,29-32,34,35,37],[39,40,64]. Click here for file
