# Supplementary material for: Similar herpes zoster incidence across Europe: results from a systematic literature review
Source: BMC Infect Dis. 2013 Apr 10;13:170. doi: 10.1186/1471-2334-13-170 (PMC3637114; doi:10.1186/1471-2334-13-170)
Supplement: Additional file 1 — Incidence rates of herpes zoster (HZ) by age group and by sex when available (/1 000) [20,22,23,25,29-32,34,35,37],[39,40,64]. [file 1471-2334-13-170-S1.doc]

**Additional file 1 -** **Incidence rates of herpes zoster (HZ) by age group and by sex when available (/1 000)**

| **Country** | **Ref.** | **Study population (All/M/W)** | **HZ incidence rates (/1 000)** | | | | | | | | | | | | | | | | | | | | | | |
| --- | --- | --- | --- | --- | --- | --- | --- | --- | --- | --- | --- | --- | --- | --- | --- | --- | --- | --- | --- | --- | --- | --- | --- | --- | --- |
|  |  |  | **Overall** | **By sex** | | **By age group (years)** | | | | | | | | | | | | | | | | | | | |
|  |  |  |  | **M** | **W** | **0-4** | **5-9** | **10-14** | **15-19** | **20-24** | **25-29** | **30-34** | **35-39** | **40-44** | **45-49** | **50-54** | **55-59** | **60-64** | **65-69** | **70-74** | **75-79** | **80-84** | **85-89** | **90-94** | **≥95** |
| **Belgium** | [20] | All | 4.57 | 4.27 | 4.84 |  |  |  |  |  |  |  |  |  |  |  |  |  |  |  |  |  |  |  |  |
|  |  | M | 4.27 |  |  | 0.8 | 3.6 | | 3.7 | | 2.9 | | | | 5 | | | | 8.8 | | 7.9 | | | | |
|  |  | W | 4.84 |  |  | 0.8 | 5.0 | | 3.1 | | 2.9 | | | | 6.4 | | | | 8.5 | | 8.4 | | | | |
| **France** | [23] | All ≥50 | 8.99 | 7.55 | 10.17 |  |  |  |  |  |  |  |  |  |  | 5.84 | | 8.39 | | 12.43 | | 13.47 | | | |
|  |  | M ≥50 | 7.55 |  |  |  |  |  |  |  |  |  |  |  |  | 5.47 | | 8.19 | | 9.07 | | 11.62 | | | |
|  |  | W ≥50 | 10.17 |  |  |  |  |  |  |  |  |  |  |  |  | 6.21 | | 8.57 | | 14.91 | | 14.37 | | | |
|  | [22] | All | 3.82 |  |  | 1.84 | | | 1.57 | | 1.74 | | 1.69 | | 4.16 | | 5.77 | | 8.68 | | 9.85 | | 10.77 | | 14.37 |
| **Germany** | [25] | All ≥50 | 9.80 | 8.00 | 11.20 |  |  |  |  |  |  |  |  |  |  | 6.80 | 6.80 | 9.60 | 8.50 | 12.30 | 12.40 | 12.50 | | | |
|  |  | IC ≥50 | 9.50 | 7.40 | 11.10 |  |  |  |  |  |  |  |  |  |  | 6.70 | 7.00 | 9.20 | 8.30 | 11.80 | 11.90 | 12.70 | | | |
| **Italy** | [29] | All ≥15 | 4.31 | 3.82 | 4.75 |  |  |  | 1.71 | 1.82 | 1.91 | 2.25 | 1.95 | 2.51 | 3.06 | 4.15 | 5.63 | 6.90 | 7.11 | 8.22 | 8.56 | 7.97 | 6.13 | | |
|  |  | IC* All ≥15 | 4.07 |  |  |  |  |  | 1.67 | 1.82 | 1.84 | 2.26 | 1.93 | 2.43 | 3.04 | 4.00 | 5.47 | 6.61 | 6.75 | 7.68 | 8.06 | 7.69 | 5.85 | | |
|  | [30] | All ≥14 | 1.74 | 1.60 | 1.87 |  |  |  | 0.63 | | | | | | 1.15 | | | | 2.45 | | 5.78 | | | | |
| **Netherlands** | [31] | All | 3.20 | 2.50 | 3.90 |  |  |  |  |  |  |  |  |  |  |  |  |  |  |  |  |  |  |  |  |
|  |  | M | 2.50 |  |  | 1.66 | | | 2.18 | | 1.42 | | | | 3.20 | | | | 5.17 | | 7.80 | | | | |
|  |  | W | 3.90 |  |  | 2.19 | | | 1.71 | | 2.18 | | | | 6.33 | | | | 5.88 | | 8.57 | | | | |
|  | [32] | All | 3.25 |  |  | 0.97 | 1.70 | 1.55 | 1.87 | 1.77 | 1.85 | 2.27 | 1.90 | 3.37 | 3.35 | 3.90 | 5.47 | 6.57 | 6.45 | 7.45 | 7.20 | 7.75 | 8.35 | | |
| **Spain** | [35] | All | 4.15 | 3.55 | 4.75 | 1.14 | 2.55 | 2.73 | 1.84 | 1.98 | 1.61 | 1.84 | 1.89 | 2.28 | 3.39 | 5.86 | 6.64 | 8.41 | 9.05 | 9.60 | 8.99 | | | | |
|  | [34] | All ≥14 | 4.10 | 2.70 | 4.50 | 1.30 | | | | | | | | | | 6.70 | | 5.20 | | 11.10 | | | | | |
| **UK** | [37] | IC ≥50 | 5.23 | 4.30 | 6.05 |  |  |  |  |  |  |  |  |  |  | 3.44 | 4.08 | 4.90 | 5.96 | 6.34 | 7.09 | 7.29 | 6.22 | | |
|  |  | M ≥50 | 4.30 |  |  |  |  |  |  |  |  |  |  |  |  | 2.50 | 3.20 | 4.00 | 5.20 | 5.80 | 6.30 | 6.40 | 5.40 | | |
|  |  | W ≥50 | 6.05 |  |  |  |  |  |  |  |  |  |  |  |  | 4.40 | 5.00 | 5.80 | 6.70 | 6.80 | 7.70 | 7.80 | 6.60 | | |
|  | [43] | All |  |  |  | 0.76 | 1.79 | | 2.10 | | | | | | 4.70 | | | | 7.71 | | | | | | |
|  | [40] | All | 3.73 |  |  | 0.92 | 2.19 | | 2.12 | | | | | | 7.12 | | | | 9.32 | | | | | | |
|  | [39] | M |  |  |  | 1.62 | | | 2.13 | | 1.99 | | | | 3.99 | | | | 7.75 | | 9.58 | | | | |
|  |  | W |  |  |  | 2.31 | | | 2.11 | | 2.45 | | | | 5.91 | | | | 9.84 | | 11.04 | | | | |

*Abbreviations: M* Men, *W* Women, *IC* Immunocompetent, *UK* United Kingdom.
